# Rho GTPases in Gynecologic Cancers: In-Depth Analysis toward the Paradigm Change from Reactive to Predictive, Preventive, and Personalized Medical Approach Benefiting the Patient and Healthcare

**DOI:** 10.3390/cancers12051292

**Published:** 2020-05-20

**Authors:** Pavol Zubor, Zuzana Dankova, Zuzana Kolkova, Veronika Holubekova, Dusan Brany, Sandra Mersakova, Marek Samec, Alena Liskova, Lenka Koklesova, Peter Kubatka, Jan Bujnak, Karol Kajo, Milos Mlyncek, Frank A. Giordano, Olga Golubnitschaja

**Affiliations:** 1 Department of Gynecologic Oncology, The Norwegian Radium Hospital, Oslo University Hospital, 0379 Oslo, Norway; prof.pavol.zubor@gmail.com; 2 OBGY Health & Care, Ltd., 01001 Zilina, Slovak Republic; 3 Biomedical Center Martin, Jessenius Faculty of Medicine in Martin, Comenius University in Bratislava, 03601 Martin, Slovak Republic; zuzana.dankova@uniba.sk (Z.D.); zuzana.snahnicanova@uniba.sk (Z.K.); veronika.holubekova@uniba.sk (V.H.); dusan.brany@uniba.sk (D.B.); sandra.mersakova@uniba.sk (S.M.); 4 Department of Obstetrics and Gynecology, Martin University Hospital and Jessenius Faculty of Medicine in Martin, Comenius University of Bratislava, 03601 Martin, Slovak Republic; marek.samec@uniba.sk (M.S.); alenka.liskova@gmail.com (A.L.); koklesova5@uniba.sk (L.K.); 5 Department of Medical Biology, Jessenius Faculty of Medicine, Comenius University in Bratislava, 03601 Martin, Slovak Republic; peter.kubatka@uniba.sk; 6 Department of Oncogynecology, Kukuras Hospital, 07101 Michalovce, Slovak Republic; janbujnak@hotmail.com; 7 Department of Pathology, St. Elizabeth Cancer Institute, 81250 Bratislava, Slovak Republic; karol.kajo@ousa.sk; 8 Department of Obstetrics and Gynecology, Faculty Hospital Nitra, Constantine the Philosopher University, 949 74 Nitra, Slovak Republic; mlyncekmilos@hotmail.com; 9 Department of Radiation Oncology, University Hospital Bonn, Rheinische Friedrich-Wilhelms-Universität Bonn, 53113 Bonn, Germany; frank.giordano@ukbonn.de; 10 Predictive, Preventive and Personalised (3P) Medicine, Department of Radiation Oncology, University Hospital Bonn, Rheinische Friedrich-Wilhelms-Universität Bonn, 53113 Bonn, Germany; 11 Breast Cancer Research Centre, UKB, Rheinische Friedrich-Wilhelms-Universität Bonn, 53113 Bonn, Germany; 12 Centre for Integrated Oncology, Cologne-Bonn, Rheinische Friedrich-Wilhelms-Universität Bonn, 53113 Bonn, Germany

**Keywords:** predictive preventive personalized medicine (PPPM/3PM), paradigm change, Rho GTPases, structure, function, regulation, signaling pathways, molecular cellular tissue level, impairments, healing, inflammation, tumorigenesis, gynecologic oncology, endometrial cancer, breast cancer, cervical cancer, ovarian cancers, metastatic disease, biomarker-set, patient stratification, in-depth diagnostics, therapy resistance, prognosis

## Abstract

Rho guanosine triphospatases (GTPases) resemble a conserved family of GTP-binding proteins regulating actin cytoskeleton dynamics and several signaling pathways central for the cell. Rho GTPases create a so-called Ras-superfamily of GTPases subdivided into subgroups comprising at least 20 members. Rho GTPases play a key regulatory role in gene expression, cell cycle control and proliferation, epithelial cell polarity, cell migration, survival, and apoptosis, among others. They also have tissue-related functions including angiogenesis being involved in inflammatory and wound healing processes. Contextually, any abnormality in the Rho GTPase function may result in severe consequences at molecular, cellular, and tissue levels. Rho GTPases also play a key role in tumorigenesis and metastatic disease. Corresponding mechanisms include a number of targets such as kinases and scaffold/adaptor-like proteins initiating GTPases-related signaling cascades. The accumulated evidence demonstrates the oncogenic relevance of Rho GTPases for several solid malignancies including breast, liver, bladder, melanoma, testicular, lung, central nervous system (CNS), head and neck, cervical, and ovarian cancers. Furthermore, Rho GTPases play a crucial role in the development of radio- and chemoresistance e.g. under cisplatin-based cancer treatment. This article provides an in-depth overview on the role of Rho GTPases in gynecological cancers, highlights relevant signaling pathways and pathomechanisms, and sheds light on their involvement in tumor progression, metastatic spread, and radio/chemo resistance. In addition, insights into a spectrum of novel biomarkers and innovative approaches based on the paradigm shift from reactive to predictive, preventive, and personalized medicine are provided.

## 1. Introduction

### 1.1. Rho GTPase Characteristics and Classification

Rho guanosine triphospatases (GTPases) are small signaling, GTP binding proteins forming a family of the Ras superfamily. The first members of the Rho GTPase family were discovered in 1985 [1]. They are evolutionary conserved and found in almost all eukaryotes. Rho GTPases are molecular switches that control signal transduction pathways and influence several cellular functions [2,3].

**Figure 1:**
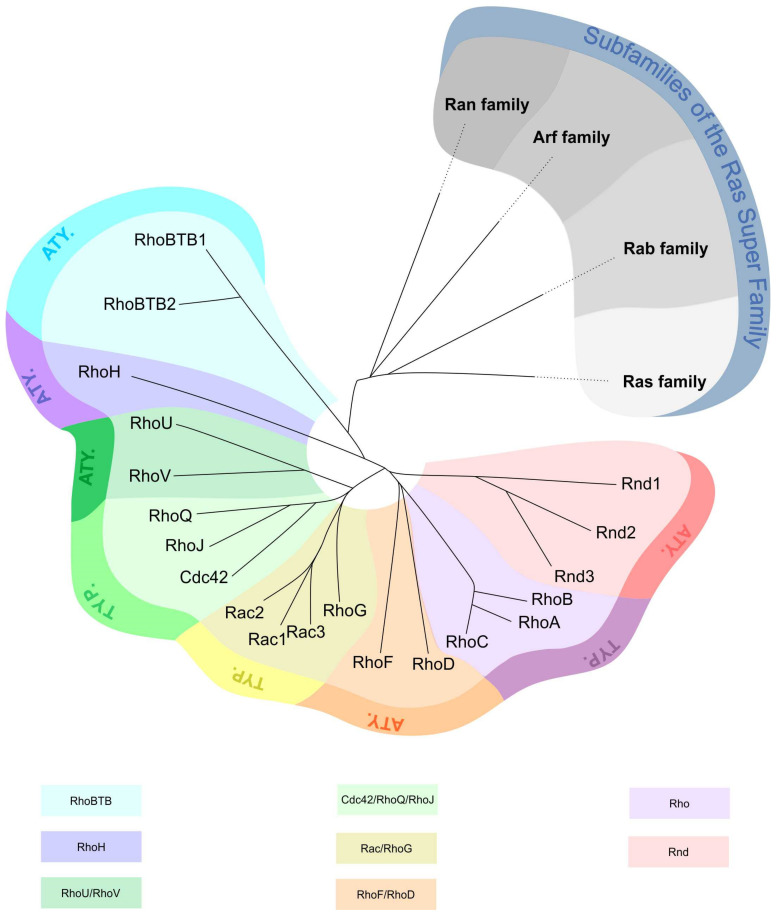
Graphical interpretation of the unrooted phylogenetic tree of Rho guanosine triphospatases (GTPases) and their connection to other Ras-like families. According to amino acid sequences, the 20 members of the family are grouped into eight subfamilies (Rac/RhoG, Rho, Cdc42/RhoQ/RhoJ, RhoF/RhoD, RhoV/RhoU, RhoH, Rnd, and RhoBTB) presented in different colors. Abbreviations: TYP., typical Rho GTPases; ATY., atypical Rho GTPases.

The 20 members of the human Rho GTPase family are divided into eight subfamilies: Cdc42 (Cdc42, RhoJ, RhoQ), Rac (Rac1, Rac2, Rac3, RhoG), RhoUV (RhoU, RhoV), Rho (RhoA, RhoB, RhoC), RhoBTB (RhoBTB1, RhoBTB2), RhoDF (RhoD, RhoF), RhoH (RhoH1), and Rnd (Rnd1, Rnd2, Rnd3) [4] (Figure 1). Based on their structure and regulation mechanism of their enzymatic activity, Rho GTPases are classified as typical and atypical [5,6]. Three typical subfamilies (Rac/RhoG, Rho, and Cdc42/RhoQ/RhoJ) act as molecular switches. They cycle between an active/inactive GTP/guanosine-5′-diphosphate (GDP)-bound forms and are regulated by several factors: guanine nucleotide exchange factors (GEFs), GTPase-activating proteins (GAPs), and guanine nucleotide dissociation inhibitors (GDIs). The other five subfamilies are atypical (RhoV/RhoU, RhoF/RhoD, RhoH, Rnd, and RhoBTB) as they are predominantly GTP-bound and most likely not regulated by GEFs or GAPs [2].

Most human Rho GTPases are characterized by ubiquitous expression, but some of them such as Rac2 and RhoH have tissue-specific expression patterns in hematopoietic cells [7].

**Figure 2:**
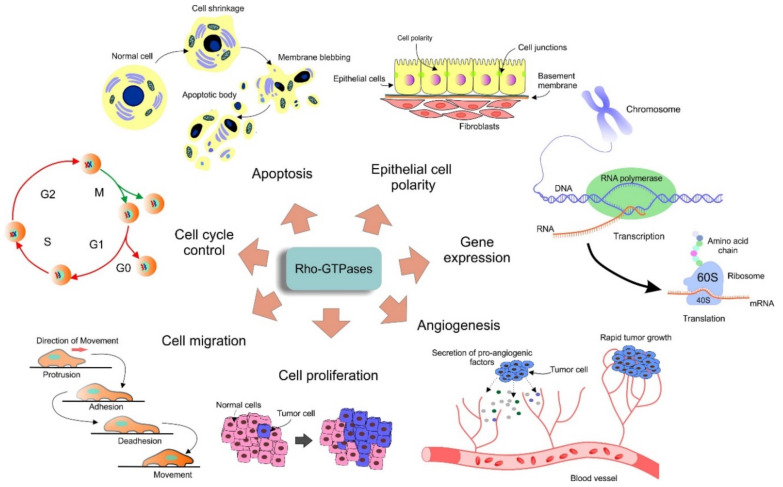
A detailed overview of Rho GTPases participating in modulation of various cellular events. Rho family members play a vital role in regulation of processes including programmed cell death, cell cycle, cell migration, epithelial cell polarity, gene expression, proliferation or angiogenesis. Disequilibrium of GTPases has a significant impact on the carcinogenesis.

Rho GTPases are part of signal cascades with roles in the regulation of various cellular processes, mainly cytoskeletal organization and dynamics, cell shape, polarity, motility, vesicular trafficking, cell division, gene expression, and others [8]. They are activated in response to extracellular stimuli in the form of soluble molecules, adhesive interactions or mechanical stresses. The change into the active GTP-bound form is accompanied by a conformational reorganization (in typical Rho GTPases) that increases the ability of Rho GTPases to bind and activate effectors like protein kinases and scaffold/adaptor-like proteins [2,5,9]. Deregulation of the Rho GTPase function is involved in various pathological processes including tumorigenesis (Figure 2) [10].

### 1.2. Rho GTPases Structure

**Figure 3:**
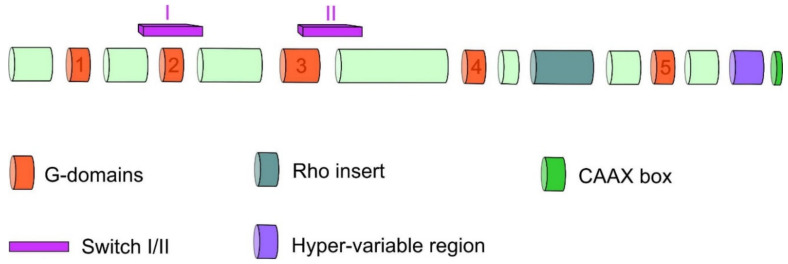
Domain structure of Rho GTPase proteins. The protein size is approximately 21–25 kDa containing the G domain (guanosine-5′-diphosphate (GDP)/GTP binding region) consisting of five conserved motifs (G1–G5), switch I and II domains, and the insert region. The ending region represents the hyper-variable region followed by the CAAX box.

Rho GTPase proteins consist of the N-terminal, G domain, Rho insert region as well as switch I and switch II regions CAAX box in the C-terminal (Figure 3) [11,12,13,14,15]. The structural core of Rho GTPases, like in other members of the Ras superfamily, is formed by G-domain containing binding side for guanine nucleotide [11]. This domain consists of five α helices surrounding six-stranded β sheets and contains five conserved motifs G1–G5 which play a role in binding and hydrolysis of guanine nucleotide and also mediate the interaction with effectors [12]. Another characteristic structure of Rho GTPases is the insert region of 13 residues with α helical conformation located between motifs G4 and G5. The inserted region participates in GEF binding as well as binding and activation of some effector proteins [13]. The C-terminal region of GTPases is composed of the short hypervariable region containing protein binding sides for specific regulatory or effector proteins [12]. The hypervariable region is followed by a conserved CAAX box where C represents cysteine, A is an aliphatic amino acid, and X means any amino acid. The CAAX box is a necessary signal for posttranslational modification (prenylation) of Rho GTPases and their subsequent localization to the cell membrane [16,17].

**Figure 4:**
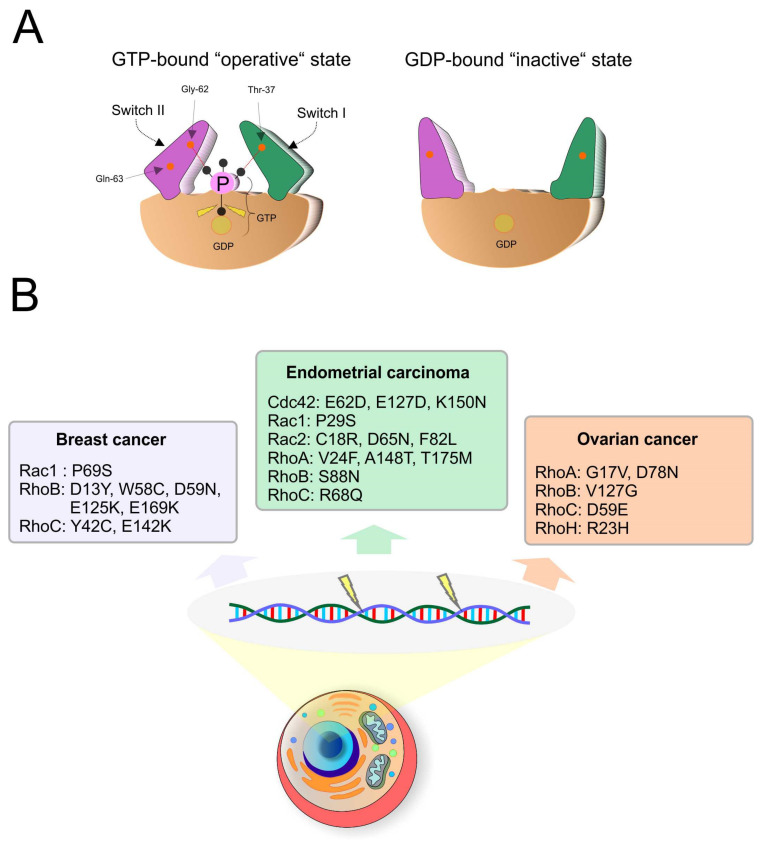
Switching mechanism of Rho GTPases. (**A**) The process of activation and inactivation of Rho enzymes is possible via structural rearrangements in at least two regions of the protein. In the operative “on” state, domains switch I (green) and switch II (purple) are bound to the γ-phosphate of GTP (pink) via interaction with conserved glycine and threonine. During GTP binding state, the two domains are in a conformation when they can interact and stimulate specific downstream effector proteins. Release of the phosphate by hydrolysis and GDP generation (yellow circle) causes switch I and switch II to relax into another conformation, the inactive “off” state incapable of binding effectors. The lower figure (**B**) describes selected mutations in Rho GTPases that were identified in human patient samples with gynecological malignancies. Abbreviations: GTP, guanosine-5′-triphosphate; GDP, guanosine-5′-diphosphate; Gln, glutamine; Gly, glycine; Thr, threonine; P, γ-phosphate.

Very important structural parts of Rho GTPases are switch I and switch II regions, which are responsible for GTP or GDP molecule binding and modulate the conformation of these regions accordingly. The highly conserved amino acids most critical for binding of GTP are threonine in position 37 (switch I) and glycine in position 62 (switch II) in RhoA. Amide groups of these amino acids create hydrogen bonds with the oxygen of the γ-phosphate group presented only in GTP. In active GTP-bound state, switch domains create conformation that allows to bind and activate downstream effectors. During GTP hydrolysis, hydrogen bonds are disrupted, and the conformation of switch domains is relaxed into an inactive state unable to bind effector proteins. Hence, the mechanism of conformation changes of switch regions is key for recognition of active and inactive states of Rho GTPases by regulatory and effector proteins (Figure 4A). Switch II region contains conserved glutamine in the 63 position in all Rho GTPase members. It is essential for GAP-catalyzed GTP hydrolysis. Mutational changes of this residue to leucine or alanine lead to constitutive activation of Rho GTPases [11,12,16,18,19]. Moreover, Figure 4B summarizes individual mutation in Rho GTPases detected in patients with gynecological malignancies [20].

The members of Rho family have a high amino acid sequence and structural homology. The most similar are RhoA, RhoB and RhoC members with 88% homology. Differences in primary sequence contribute to divergent surface charge. It leads to selective binding of regulators (GAPs, GEFs, GDIs) and effectors and to specific downstream signaling. The roles and actions of Rho GTPases depend also on cell localization and cellular context [12]. RhoBTB (bric à brac) 1 and 2 differ from other Rho GTPases. They possess two broad-complex, tramtrack, bric à brac (BTB) domains that mediate homodimerization and heterodimerization of RhoBTBs and are involved in protein–protein interactions [21]. Their GTP-binding domain lacks conserved amino acids essential for GTP hydrolysis, therefore it is assumed they are not able to hydrolyze GTP [21,22], nor are they able to promote actin filament organization [23]. On the other hand, there is evidence that they are involved in the ubiquitin ligase complex [21,24].

### 1.3. Rho GTPases Function

The Rho GTPases have roles in signaling pathways regulating cellular processes, such as cytoskeletal organization, cell motility, division, and migration, amongst others. They are also involved in cancer-triggering and cancer-promoting processes such as inflammation, angiogenesis, wound repair, progression, metastasis, and therapeutic response [2,8,25,26]. They are activated after binding of extracellular ligands (mitogens, growth factors, and other soluble molecules) to cell-surface receptors like integrins, cadherins, cytokine receptors, Tyr kinase receptors or G protein-coupled receptors [27]. As soon as they adopt the active GTP-bound conformation, they are able to interact with various effector proteins and in this way stimulate downstream signaling cascade. Serine-threonine kinases, tyrosine kinases, lipid kinases, oxidases, and scaffold proteins are main effectors of Rho GTPases [28]. Depending on cell type and type of extracellular signal, certain Rho GTPases might interact with different effector proteins thereby regulating different cellular processes. For three common Rho GTPases—RhoA, Rac1 and Cdc42—more than 60 effectors have been identified [8]. For instance, activation of Rac1 as a response to epidermal growth factor (EGF) or platelet-derived growth factor (PDGF) leads to polymerization of actin fibers and lamellipodia formation [29]. Rac1 also participates in the activation of Nox family NADPH oxidase producing superoxide during antimicrobial defense [30,31]. In addition to cytoskeletal organization, Rho GTPases modulate enzymatic activities and are also part of signal pathways affecting transcriptional factors and hence the level of gene expression [28]. Considering the role of Rho GTPases in cell division, they are involved in the regulation of cell cycle initiation, attachment of microtubules to kinetochores, and in cytokinesis where they regulate contractile ring formation [29,32].

### 1.4. Rho GTPases Regulation

The Rho GTPases’ characteristic switch between a GTP-bound or GDP-bound status is regulated by various mechanisms involving regulating proteins as well as posttranscriptional and posttranslational modifications. There are three types of regulating proteins, which are also the subject of posttranslational modifications that represent another level of Rho GTPase regulation to maintain a proper equilibrium between active and inactive states of GTPases [33]. Rho GTPases are activated by replacement of GDP with GTP which is mediated by guanine nucleotide exchange factors (GEFs) that act in a positive regulatory manner. A total of 82 GEFs are divided into two families based on their structural domains: Dbl family and DOCK [27,34,35]. Most GEFs possess a catalytic DBL-homology (DH) domain and pleckstrin homology (PH) domain, therefore they are classified into the DBL family [27,36]. GEFs belonging to the DOCK family are characteristic with two conserved domains: DOCK homology region 1 and 2 (DHR 1, DHR2) [37]. Since intrinsic GTPase activity of Rho GTPases is low, the rate of GTP hydrolysis is stimulated by GTPase activating proteins (GAPs) which are considered as negative regulators of GTPase activity. There are 66 Rho GAPs in humans with conserved 150 amino acid domain which binds to Rho GTPase in the active state and increases its hydrolysis activity, leading to inactivation of Rho GTPase [38]. Another family of regulating proteins, though without enzymatic activity, are guanine nucleotide dissociation inhibitors (RhoGDI1, RhoGDI2, RhoGDI3) which play the role of chaperones [18]. They interact through their N-terminal domain with the switch domains of Rho GTPases, thereby preventing GTP binding. Another role of GDIs is the extraction of Rho GTPases from the membrane and their storage in an inactive state in cytosol; they also protect them against proteolytic degradation [39,40]. Proper localization within the cell and biological function of Rho GTPases are regulated by various posttranslational modifications including lipid modifications, phosphorylation, ubiquitination or sumoylation which do not affect GTPase activity [2,33]. Rho GTPases can also be regulated on the transcriptional level by alternative splicing or microRNA-based silencing [41,42].

**Figure 5:**
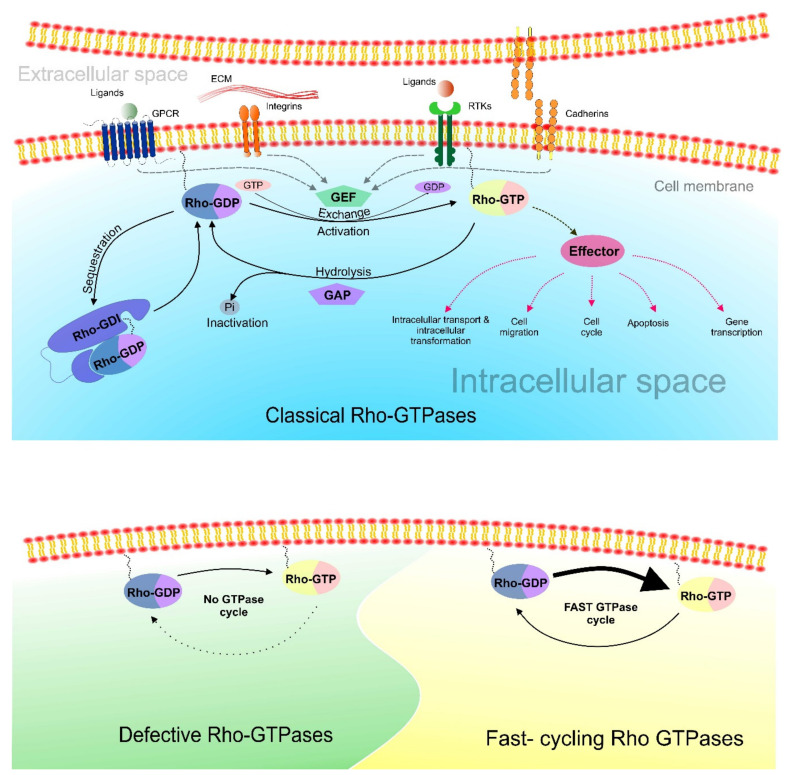
Rho GTPases regulation. GTP bound and GDP bound Rho GTPases are illustrated as blue-purple and yellow-pink circles, respectively. Positive regulator guanine nucleotide exchange factor (GEF) (green) and negative regulator GTPase-activating protein (GAP) (purple) are shown as pentagon structure between inactive and active forms of Rho GTPase. Active Rho GTPase interacts with different effector proteins (magenta). Rho-GDI (blue) keep GTPases in inactive form through their extraction from membranes. The upper image represents cycling of typical Rho GTPases and their interactions with surface receptors. The lower picture illustrates defective and fast-cycling Rho GTPases without GAP/GEF participation. Abbreviations: GTP, guanosine-5′-triphosphate; GDP, guanosine-5′-diphosphate; GEF, guanine nucleotide exchange factor; GAP, GTPase activating protein; GDI, guanine nucleotide dissociation inhibitor; ECM, extracellular matrix; GPCR, G-protein-coupled receptor; RTKs, receptor tyrosine kinases.

Unlike typical Rho GTPases, atypical Rho GTPases are constantly active in their basic GTP-bound form [2,43,44]. One class of Rho GTPases, named fast-cycling Rho GTPases (RhoU, RhoV, RhoD, RhoF), dispose of increased intrinsic nucleotide exchange activity and intact hydrolysis ability [44,45]. After GTP hydrolysis by these Rho GTPases, GDP is replaced by GTP by their own exchange activity as the intracellular concentration of GTP is 10-fold higher than GDP [46]. Another class of atypical Rho GTPases is called defective Rho GTPases (RhoH, Rnd1–3, RhoBTB1,2). These proteins exhibit an amino acid substitution in the Rho domain that negatively affects their GTPase activity that hampers proper GTP–GDP cycling and thus keeps them exclusively in the GTP-bound form. The activity of atypical Rho GTPases is modulated on an expression level and by posttranslational modifications [33]. Figure 5 summarizes the differences in regulation between classical, defective, and fast-cycling Rho GTPases.

### 1.5. The Role of GTPases in Tumorigenesis

Since Rho GTPases play roles in signaling pathways regulating important cellular processes as cell cycle and cell motility, their dysregulation might disrupt controlling of these processes and contribute to carcinogenesis and metastasis. It has been suggested, that the altered function of Rho GTPase family members in cancers is a consequence of mutations or aberrant expression pattern not only GTPases themselves but also their upstream regulators or network with other small GTPases [2,20]. As Rho GTPases activity and signaling is strictly regulated by various mechanisms, changes in any of regulatory proteins or posttranscriptional and posttranslational modifications can support proliferation, survival, and invasion of tumor cells. Overexpression of positive regulators (GEFs) as well as downregulation of negative regulators (GAPs) can lead to constitutive active Rho-dependent signalization [47]. On the other hand, downregulation of some Rho GTPases in tumor cells was also reported [10,48]. Considering the complex role of Rho GTPases in carcinogenesis, it is not surprising that Rho GTPases as well as their regulators are potential targets for cancer therapies [29].

## 2. Rho GTPases in Gynecological Cancer

As Rho GTPases have a function in basic cellular processes, their role is very important for cellular maintenance and eventual carcinogenesis. The most studied Rho GTPases in oncogynecology are RhoA, RhoC, Rac1, and Cdc42, which show higher expression correlating with the advancement of the cancer. Some Rho GTPases have the potential to be prognostic markers for tumors with a potential to metastasize. However, not all results are conclusive and more detailed studies are needed to clarify the biology of the Rho GTPases’ interplay as well as their interaction with other pathway components (Ran, Ras, and Rab GTPases, proteins, receptors and others) in the context of diagnostic and prognostic markers and advanced personalized treatment algorithm with effective therapeutic targets.

### 2.1. Ovarian Cancer

Ovarian cancer is one of the most lethal gynecological malignancies. It tends to develop from different tissues as a result of an accumulation of genetic changes on one hand and due to post-genomic modifications on the other hand [49]. Epithelial ovarian cancer (EOC) is the most common type of ovarian cancer (90%) and more than 140,000 patients succumb to this disease each year [50]. Other major types originate from stromal cells (5%) and germ cells (3%) [50]; to this end, many rare subtypes are linked to specific outcomes [51]. The poor prognosis is due to a long period of asymptomatic cancer growth with delayed detection in (very) advanced stages [45,46,47], severe comorbidity in elderly, lack of patient centralization, and differences in disease treatment management and disease biological profile [52,53,54,55,56]. Global miRNA expression analysis of serous and clear cell ovarian carcinomas identifies differentially expressed miRNAs including miR-200c-3p as a prognostic marker [57]. Therefore, a deeper understanding of the molecular process occurring at early cancer stages would allow for identifying the process-specific biomarkers and thus result in an earlier or even predictive diagnosis.

The best studied human proteins belonging to the Ras superfamily of small GTPases in ovarian cancer are Rho, Ras, and Ran.

One of the oldest Rho GTPases is the transforming protein RhoA. It is encoded by the *RHOA* gene and is in/activated by GDIs/GEFs. RhoA GTPase regulates several aspects of tumorigenesis and aberrant expression is associated with poor tumor differentiation and advanced stages of ovarian cancer. Previous studies [58,59,60] described Ran–RhoA signaling pathway, which regulates EOC invasion by RhoA stabilization at the plasmatic membrane and its activation. Their findings could lead to potential targeted drug development preventing ovarian tumor metastasis [60].

Horiuchi with colleagues [61] demonstrated that mRNA expression levels of RhoA and RhoC were significantly higher in invasive carcinomas than in benign cystadenomas. They also showed that there was no difference in the expression of the effector protein of Rho-kinase (ROCK) nor in the expression of the inhibitor for conversion among active and inactive forms of Rho (Rho GDI) between ovarian tumors. The RhoA protein also contains a CAAX and causes earmarking of specific plasma membrane (PM) microdomains [62]. Zaoui et al. [60] found out that on PM RhoA GTPase formed a complex with another small GTPase nucleocytoplasmic shuttle protein Ras-related nuclear protein (Ran) [63], which is strongly associated with EOC progression and poor overall survival [64]. This complex leads to activation of RhoA. Authors in this study demonstrated a link between Ran and RhoA signaling pathway and suggested that downregulation of Ran might affect ovarian cancer cell proliferation through a degradation of RhoA by shifting its balance. This Ran–RhoA signaling complex seems to be a possible molecular target for controlling cancer metastasis [60].

In EOC, the activation of RhoA (along with the Rac1 and Cdc42) GTPases has shown to be regulated by cytoplasmic p120 catenin (p120ctn, also known as CTNND1) [65]. p120ctn is a 120 kDa substrate of the oncogenic SRC tyrosine kinase [66] and can interact with the transmembrane glycoproteine mucin 1 MUC1 cytoplasmic domain [67] to modulate oncogenic signaling cascades. Chen et al. [67] studied a potential relationship between Mucin-16 and p120ctn. Mucin-16 (MUC16, CA125) is a type I transmembrane protein that has a critical pro-tumorigenic role in EOC [68] and shares many structural similarities with MUC1. Chen et al. [67] found out that MUC16 and p120ctn were overexpressed in ovarian cancer patients and showed that the subcellular translocation of p120ctn was impacted by MUC16 through RhoA/Cdc42 activation in EOC.

Furthermore, it has been hypothesized that genetic variations in small GTPases might be associated with a higher risk of developing EOC [69]. The Rho family includes 20 human genes, out of which three are the most well-known (*RhoA*, *Rac1*, and *Cdc42*). Rac1 and Cdc42 are highly expressed in EOC [70] and therefore inhibitors of these two GTPases have undergone research on primary human ovarian cancer cells. A published study showed that R-enantiomer of the anti-inflammatory drug ketorolac inhibited Rac1 and Cdc42 and showed a better outcome in ovarian cancer treatment [71].

ARHGEF10L is (Rho guanine nucleotide exchange factor 10 like) a GEF for RhoA. It is a member of the RhoGEF family which activates Rho GTPases [72]. Earp et al. [69] described specific single nucleotide polymorphism SNP variants in *ARHGEF10L* gene to be related with higher endometrioid EOC risk (rs2256787), and rs10788679 to be related with invasive serous EOC risk. Two other SNP variants (rs1955513, rs927062) in *AKAP6* (A-kinase anchoring protein 6) gene were associated with risk of invasive EOC. AKAP6 is associated with G protein signaling and SNPs in this gene cause expression of the Rho GTPase activating protein 5 (ARHGAP5) and negative regulation of Rho GTPases.

Rho family proteins are primarily involved in human cancers through their hyperactivation and/or overexpression but only secondary due to the point mutation activation [73] that points to their decisive role in chemotherapeutic resistance [74,75]. Cisplatin (a platinum-based anti-neoplastic drug) is one of the most common treatment options in ovarian cancer. It was discovered that cisplatin resistance of ovarian cancer cells was linked to dissimilarity in the actin cytoskeleton and stress fibers [76]. In addition, a mechanical tropism was shown. Cultured metastatic ovarian cancer cells exhibit higher resistance to chemotherapeutic drugs; the resistance mechanism involves the Rho/ROCK signaling pathway [77]. The above results strengthen the idea that mechanical signals can be a leading factor in ovarian cancer progression and chemoresistance.

### 2.2. Endometrial Cancer

Endometrial cancer (EC) is one of the most common uterine cancers with increasing occurrence in recent years [78]. Although the increasing incidence is particularly pronounced in postmenopausal women, in the last decade, a significant increase was reported for women younger than 55 years [79]. The incidence of EC is approximately 25/100,000 women in Europe and 19/100,000 women in the United States. The age-adjusted mortality in Europe is as high as 4 per 100,000 women, whereas it is even higher (4.6 per 100,000) for women in the United States [80]. EC resembles a very heterogeneous group of tumors, whose development is associated with several risk factors, mainly the presence of polycystic ovaries, nulliparity or lower parity, oral contraceptive use, tamoxifen use, lower age of menarche, African origin, high body mass index, dyslipidemia or diabetes [81,82,83,84,85]. Furthermore, EC is typical for older postmenopausal women as only 5 % of all women are diagnosed before the age of 40 and the majority of women are diagnosed after 60 years of age [86].

EC is traditionally classified by histopathological characteristics into two groups. Type I includes estrogen-dependent tumors, whose development is usually related to the previous formation of hyperplasia. This group is much more common and accounts for up to 80% of all tumors [83,87]. Type II are estrogen-independent tumors with higher risk, aggressiveness and mitotic index, lower apoptotic activity, and rapidly metastasize [83,88]. Stage, type, and aggressiveness are important factors for selection of surgical approach, which impacts on the final disease outcome [89,90].

The development of EC is also more frequent in patients with Lynch syndrome or Cowden syndrome [91,92]. Several genetic alterations are frequently observed in EC development: loss of functions, enhanced gene expression, mutations, hypermethylation as well as chromosomal aberrations such as gene fusions or deletions [81,82,83,93]. The deletion of 1p.36 locus including *MIIP* (migration and invasion inhibitory protein) gene results in lower gene expression in EC and in vitro conditions reflect the cancer stage [94]. However, more importantly, *MIIP* as a tumor suppressor attenuates Rac1 [94]. In particular, it competes with Rac1 in binding to serine-threonine protein kinase (PAK1). The interaction of Rac1 and its downstream effector PAK1 affects actin reorganization, mainly through mediation of lamellipodia protrusion formation, which subsequently affects cell motility, migration, adhesivity, and morphology [95,96]. Abnormal activity of actin cytoskeleton regulatory proteins may cause increased cancer cell mobility, contributing to faster disease progression and subsequently faster metastasis formation as well. Therefore, the faster the reorganization of the actin cytoskeleton in cancer cells, the greater their ability to invade [97,98].

Sales et al. [99] investigated the role of prostaglandin F_2α_ in mediating adhesion, morphology, and migration of well differentiated endometrial adenocarcinoma cells (Ishikawa) via the F-prostanoid receptor as well as in Rho content. The inhibition of two RhoA GTPases—Rac1 and CDC42—significantly decreased the ability of cells to adhere to vitronectin after prostaglandin treatment. Therefore, both Rho GTPases play an important role in this cell cascade, and their inhibition can be considered as a possible way to influence EC progression. Vitronectin is a glycoprotein presented in extracellular matrix, whose abnormal activity is often associated with invasion and metastasis of variable cancer [100]. In addition, prostaglandins were shown to be a risk factor for both benign and malignant tumor formation in the endometrium [101,102].

Higher activity of Rac1 and PAK1 in Ishikawa cells was demonstrated by Salker and colleagues [103]. Furthermore, they showed that after affection of these cells with a cytokine LeftyA, both Rac1 and PAK1 expression were significantly reduced, resulting in depolymerization of actin and several morphological changes. It was demonstrated that LeftyA has an effect on inhibiting proliferation and activation of apoptotic cascades, and therefore is considered a suitable anti-tumor agent [104]; it specifically acts negatively on Na^+^/H^+^ exchanger 1 (NHE1) activity [105]. Abnormal increase in NHE1 activity correlates with tumor aggressiveness and ability to spread in variable cancer types [106]. This is mainly because NHE1 increased activity leads to cell acidosis, which creates a suitable environment for cancer cell development. In addition, the effect of NHE1 on the enhancement of cell motility is also expected, although the precise mechanisms in this regard are not known [107]. Finally, it is known that regulators of NHE1 activity are two GTPase—Rac1 and RhoA [108]—which, however, act antagonistically. In deprived conditions Rac1 has a positive effect on NHE1 stimulation, while RHoA has a negative effect, and vice versa for basal conditions. Nonetheless, deprived conditions better imitate cancer-cell environment, and higher NHE1 activity was noted in such conditions [109,110]. Therefore, it is appropriate to consider the possibility that reduced Rac1 activity is tightly connected with the avoidance of NHE1 signaling pathway after treatment of Ishikawa cells with LeftyA.

It is worth nothing that Neuromedin U (NMU) had an effect on endometrial cancer cells, as previously demonstrated [111]. NMU is a neuropeptide that has several roles in the physiological state, but mainly affects smooth muscle contraction [112]. Its receptors occur naturally in uterine tissues, but at least in the case of endometrial cancer tissues under in vitro conditions, their expression should be higher and directly proportional to cancer grade [111]. Particularly, in cancer cell lines derived from grade II endometrial tumors, it has been shown that higher NMU signaling activity affects the expression of variable adhesion molecules, including integrin alpha 1. More importantly, it also increases the activity of the SRC pathway and its downstream effectors RhoA and Rac1. Therefore, two Rho GTPases—RhoA and Rac1—act as important signal transducers within the NMU-SRC-Rho GTPase axis, which affects cell adhesion, spreading, migration, and proliferation [111]. In general, crosstalk between integrins, SRC, and Rho GTPases is known as one of the major factors involved in the resulting cell adhesion as well as other interactions between cells and extracellular matrix [113]; in addition, the effect of integrins to onset and spreading of EC has been proven [114]. Therefore, investigating the cascade of reactions between integrins and Rho GTPases could be beneficial for a better understanding of the origin and especially the spread of EC.

Dysregulation of RhoA GTPases pathways can be potentially affected also by abnormal activity of miRNAs, especially miRNA-204. It has been shown to be typically downregulated in endometrial cancer [115,116]. Furthermore, in renal, ovarian, and breast cancer lines, loss of miRNA-204 activity caused an increase of *BDNF* gene expression. It subsequently led to the activation of the AKT/mTOR cascade, which also includes Rac1 RhoA GTPase. After this activation, actin fibers reorganization and cell invasion ability increased [117]. Although this analysis has not been performed on endometrial cell lines, it cannot be ruled out that a similar mechanism happens in endometrial cancer cells. This assumption is based on the fact that *DBFD* gene has been shown to be one of the essential factors for endometrial proliferation and estrogen effector [118]. Therefore, abnormally increased activity of *BDNF* gene due to the loss of miRNA-204 activity may result in abnormal endometrial cell proliferation, in which Rac1 GTPase significantly participates. However, appropriate analyses would need to be performed for final evaluation of this hypothesis. In addition, although the possibility that change in Rho GTPase activity in EC occurs due to mutation changes cannot be completely ruled out, no case of any RhoA GTpase mutation in endometrial cancer has been reported to date [93,119,120].

Abnormal activity of RhoA/ROCKII signaling pathway was demonstrated in eutopic endometrial stromal cells of patients with endometriosis [121]. A higher RhoA activity in cell cultures from endometriosis lesions was also observed in a later study by Wu et al. [122]. Furthermore, Jiang et al. [123] showed that the expression of RhoA and both of its downstream effectors, ROCKI and ROCKII, was lowest in healthy myometrium, higher in eutopic endometrium, and highest in ectopic endometrium in women with adenomyosis. In addition, this higher expression level of RhoA and its downstream effectors was in direct proportion to severity of menorrhagia. In addition, the level of the protein products of these genes was the lowest in healthy myometrium. Lastly, higher RhoA/ROCKI pathway activity in women with adenomyosis was also demonstrated in a study by Wang et al. [124]. Interestingly, this study noted that in healthy women, RhoA/ROCKI activity varied relative to the menstrual cycle phase but was increased permanently in women with adenomyosis.

### 2.3. Cervical Cancer

Cervical cancer still remains the fourth most common cancer in women worldwide. Even among women aged 15–45 years, it is one of the top three cancers in 146 countries. In 2018, almost 570,000 women developed cervical cancer and 311,000 women died from it. The global incidence and mortality of cervical cancer is approx. 13.1 and 6.9 per 100,000 women, respectively and both rates depend on age [125].

Cervical cancer is a preventable disease and its incidence could be minimized by effective introduction of cervical cancer screening, management of detected disease, and vaccination of young girls [126]. The screening of cervical cancer is based on cytology examination of exfoliated cervical epithelial cells or human papillomavirus (HPV) testing, where HPV-based approaches also allow self-sampling [127,128]. In suspicious lesions, colposcopy and biopsy removal with histological examination are recommended. According to morphological changes of cells, samples are divided into several grades of cervical intraepithelial neoplasia (CIN1-3) and/or carcinoma in situ (CIS) or invasive cancer. CIN1 and CIN2-3 represent low-grade (LSIL) and high-grade squamous intraepithelial lesions (HSIL) in cytological classification, respectively [129]. As the whole strategy of surgical and adjuvant treatment for invasive cancer is changing with the advancement in radio- and chemotherapy [130,131], the primary scope for science is to understand the biology of origin and therapeutic response for this malignancy.

Small GTPases of the Rho family have been studied mainly on cervical cancer cell lines and RhoA, Rac1, and Cdc42 are the best characterized. Rho GTPAses are responsible for regulation of cell motility and organization of actin cytoskeleton.

From the well-described Rho GTPases, a high RhoA expression was found in vitro and in vivo cervical cancer tissue. In vitro experiments showed that RhoA overexpression together with ROCK1 and ROCK2 supported spreading and migration of HeLa cells, meaning a higher metastatic potential of cancer cells [132]. Nuclear localization of Rac1 was found in low-grade squamous intraepithelial lesions (LSIL) and high-grade squamous intraepithelial lesions (HSIL), as well as in tumor cells SiHA (HPV-16 infected) and C33A (HPV-negative). Cytoplasmatic location of Rac1 was found in human immortalized keratinocytes and inhibition of Rac1 expression reduced cellular proliferation [133]. Overexpression of Cdc42 GTPase was found in LSIL and HSIL and might be associated with progression of cervical lesion to cancer [134]. In vitro experiments on HeLa cells derived from human cervical carcinoma helped to elucidate some interactions of Rho GTPases and cell behavior. HeLa cells expressing the Rho GTPases as Rac1 and Cdc42 have limited the elongation ability that is maintained or reinforced by co-expression of DOCK10 protein (GEF family) and GTPase Cdc42. An increased spreading ability of cells expressing DOCK10 with non-elongated morphology is mediated mainly through Rac1 GTPase. The results suggest that DOCK10/Cdc42 cells exhibit a large amount of filopodia and decreased membrane ruffling. On the other side, DOCK10/Rac1 cells were observed with membrane ruffling and reduction of filopodia [135]. It is also speculated that RhoG GTPase participates on the adjustment of Rac function in migrating cells [136]. Not all members of Rho family show GTPase activity. The atypical members RhoBTB1 and RhoBTB2 obviously act as transcriptional regulators of hemostatic chemokine CXCL14 expression in normal epithelial cells, as well as in Hela cells [137].

Epithelial to mesenchymal transition (EMT) is frequently mentioned in connection with metastatic cancer, including cervical malignancy. As the Rho GTPases regulate the organization of the cytoskeleton, it is known that RhoC regulates the arrangement of actins. Inhibition of RhoC and Notch1 resulted in elimination of fibronectin expression and actin stress fiber formation, two relevant changes linked to EMT [138]. In some studies, using cervical carcinoma models, EMT mediated by tumor growth factor-beta (TGF-β) was reported. Rho GTPases have various function in TGF-β driven EMT (e.g., the requirement of RhoC overexpression) [139]. On the other side, RhoE suppressed TGF-β driven EMT in part through RhoA GTPase and ROCK inhibitor signaling [140]. These studies lead to the idea that GTPases could use different cellular pathways to EMT and therefore more research is needed.

Cervical cancer is mainly caused by chronic infection with high-risk human papillomavirus (hr-HPV). The oncogenic potential of hr-HPV is attributed to its E6 and E7 oncoproteins. E6 can inactivate tumor suppressor protein 53 (p53) and E7 binds to the retinoblastoma protein (PRB) and causes its destabilization [141]. The activation of well-described Rho GTPases (RhoA, Rac1, and Cdc-42) and increased cell motility were found in HPV16-infected cervical cell lines CaSki and SiHa when compared to immortalized keratinocytes (C-33A) that were HPV-negative [142]. The presence of HPV16 E6 oncoprotein was associated with a higher expression of Rho regulatory protein ARHGEF16 and enhanced activation of Cdc42 in non-transformed human keratinocytes (hTert cells) [143]. Another regulatory protein RhoG-specific guanine nucleotide exchange factor, ARHGEF26 (SGEF), was reported to interact with human discs large (DLG1) tumor suppressor and viral E6 oncoprotein in HPV16 and HPV18 positive cell lines. Therefore, E6 maintains a high expression of RhoG GTPase and increased invasive potential of cells [144]. HPV E7 is involved in metastasis and cell migration through regulation of RhoA and Rac1. RhoA GTPase is negatively regulated by ARHGAP35 (p190GAP family), which is one of the E7 targets. Dysregulation of ARHGAP35 leads to changes in actin cytoskeleton and cellular properties that could be important for viral life cycle and cell transformation [145]. Primary keratinocytes expressing HPV16 E7 oncoprotein migrated more rapidly in the hand of AKT-dependent pathway and decrement of RhoA expression [146]. Rho GTPases are probably important for viral infection and its life cycle. Elucidation of Rho GTPase signaling and interactions with viral proteins still remain under investigation.

Understanding viral protein interactions with Rho GTPase signaling networks might help in the development of therapeutic drugs. Most studies have been performed utilizing in vitro models. A study showed that Rac1 plays an important role in the regulation of apoptosis in cervical cancer cells, and its reduction can potentially increase chemosensitivity to cisplatin [147]. Under experimental conditions on HeLa cells, the authors found that a phytochemical quercetin decreased Rac1 expression, which could be useful in reduction of cervical cancer metastasis [148]. Similarly, the effect of anti-cancer phytochemical rocaglamide-A inhibited the activity of RhoA, Rac1, and Cdc42 GTPases and suppressed metastasis formation [149].

Increased radio sensitivity of HeLa cells has been described by the deterioration of DNA damage repair through modulation of RhoA GTPase activity. Contextually, a combination of chemotherapy containing RhoA inhibitors and radiotherapy might be relevant for various stages of cervical cancer development [150]. RhoC GTPase together with its downstream regulator ROCK2 regulate the radiation response and supply the radio resistance in cervical cancer. Inhibition of ROCK2 therefore could be useful in sensitization of tumor cells to irradiation [151].

### 2.4. Breast Cancer

Breast cancer (BC) is a multifactorial disease [152] and the most common cause of cancer death in women worldwide [153]. Globally, it is estimated that yearly there were 1.7 million new diagnoses from invasive breast cancer [154]. The most important factors affecting the incidence of BC are genetic, environmental, and lifestyle aspects [155]. In addition, metastatic spread is crucial in higher mortality [156].

Rho GTPases (RhoA, Rac1, and Cdc42) have been studied in connection with breast carcinogenesis, as well as in connection with other cancers, for many years [2,157]. However, the role of RhoA GTPase is still unclear. Earlier studies suggested a possibility of RhoA as an oncogenic marker. Recent studies suggest otherwise [158,159]. Some studies show potential tumor and metastasis suppressive ability of RhoA in breast cancer [160,161]. Kalpana et al. (2019) demonstrated that reduced RhoA expression increases BC lymph nodes and lung metastasis by promoting a pro-tumor microenvironment, with increased macrophage infiltration [162]. Another study showed that RhoA and RhoC GTPases regulate M2a macrophages as the strongest inducers of BC invasion. These findings point to possible uses of Rho GTPases for BC metastases prevention [156].

Cdc42, a molecular switch, is mainly involved in the regulation of the actin cytoskeleton and is often overexpressed in BC cells. Another role of Cdc42 might be as an inhibitor of tumor-suppressor genes, like *p53*, to relieve antiproliferation or apoptosis effects [163].

Yadav and colleagues (2019) indicated that expression of Rac1 and RhoA GTPases protein markers was significantly higher in BC cells then in control samples. After stretching for a longer period of time, the malignant cells increased accuracy of markers and their overexpression [164].

## 3. A Role of Rho GTPases in Cancer Therapy

Studies on aberrant Rho GTPase signaling activities have pointed to an important association with several cancer types. Key components of these pathways, such as ROCK, Rho GTPases and its regulators GAPs, GEFs, and GDIs are very interesting and promising targets for therapy. However, the manipulation and inhibition of the components require a lot of attention, as most Rho GTPases play an important role in fundamental cellular mechanisms and side effects could have serious consequences.

### 3.1. ROCK Inhibitors

Targeting the major downstream effector of RhoA, Rho-kinase (ROCK) is a promising cancer therapeutic approach [165,166]. Inhibition of Rho/ROCK pathway attenuates tumor invasion and metastasis in many in vitro and in vivo cancer models [166,167]. In addition, ROCK inhibitors such as AT13148 did not induce tumor regression in in vivo cancer studies [168].

#### 3.1.1. Y-27632

A pyridine-analog, small molecule Y-27632, was identified in 1997 by Uehata and colleagues [169]. Y-27632 inhibited ROCK1 in human cervical carcinoma (CaSki) cells with overexpressed RhoC that eventually led to decreases in the rate of invasiveness and migration. Blockage of the RhoC downstream signaling by small molecule inhibitors may represent a potent treatment strategy to inhibit tumor invasion and migration [170].

Cervical cancer metastasis is promoted by VEGF-C upregulating and activating moesin protein through RhoA/ROCK-2 pathway. Eventually, Y-27632 inhibited ROCK-2 and therefore impaired VEGF-C-induced moesin expression and phosphorylation in cervical carcinoma (SiHa) cell line [171]. Similarly, pre-treatment with an inhibitor of RhoA (C3) and Y-27632 led to the decrease in leptin-induced expression of urokinase plasminogen activator and inhibited the invasiveness of ovarian cancer (OVCAR3, SKOV-3, CAOV-3) cells [172]. Similarly, pre-treatment with Y-27632 or overexpression of a dominant-negative mutant of Rho inhibited lysophosphatidic acid (LPA) proteolytic enzyme expression and invasiveness of ovarian cancer (CAOV-3, PA-1) cells [173]. Moreover, RhoA activators, LPA, and calpeptin increased cell proliferation in endometrial carcinoma (HEC-1A) cells. The treatment with Y-27632 inhibitor reversed the effect on LPA and calpeptin-induced HEC-1A cell proliferation. However, Y-27632 increased HEC-1A cell proliferation in the absence of RhoA activators [174].

#### 3.1.2. Fasudil

Fasudil, a known ROCK-specific inhibitor that competes with ATP for binding to the kinase, was developed for the treatment of cerebral vasospasm; however, it has not been approved by U.S. Food and Drug Administration (FDA) and European agencies [175]. Fasudil decreased invasiveness and motility of human ovarian cancer cells through inhibition of LPA/Rho/ROCK pathway. In addition, Fasudil caused the loss of intracellular cytoskeletal rearrangement (stress fiber formation and focal adhesion assembly), inhibited tyrosine phosphorylation of paxillin (important focal adhesion molecule), and suppressed serine phosphorylation of myosin light chain (necessary for cell migration). In the same study, Fasudil decreased tumor burden and ascites formation in SKOV-3ip1 ovarian cancer xenografts [176]. Rho/ROCK pathway inhibition induced by Fasudil has been documented as an effective approach against viability of human ovarian cancer cells. In this regard, Fasudil increased cisplatin-induced growth inhibition and apoptosis via the modulation of hypoxia-inducible factor-1α (HIF-1α) signal transduction in A2780 cells [166].

#### 3.1.3. Phytochemicals

There are only limited data pointing to anti-cancer properties of naturally occurring substances via ROCK signaling. Yin et al. demonstrated the dose-dependent pro-apoptotic efficacy of curcumin in SKOV-3 ovarian cancer cells partly due to the activation of RhoA/Rho-kinase signaling, which may, in part, explain some postulated beneficial effects of curcumin in the therapy of ovarian cancer [177]. In another study, resveratrol suppressed interleukin (IL)-6-induced cell migration in OVCAR3 ovarian cancer lines via the downregulation of *ARH-I* (encodes Ras homolog GTPase of 26 kD) and the inhibition of LC3-positive autophagic vacuoles formation [178].

### 3.2. RhoA, Rac1, and Cdc42 Inhibitors

#### 3.2.1. Ketorolac

Ketorolac was found in two enantiomers, specifically S- and R-enantiomers. S-ketorolac is a potent and non-selective cyclooxygenase COX inhibitor and is inactive against GTPase targets. On the contrary, R-ketorolac is a noncompetitive inhibitor of Rac1 and Cdc42 and is inactive against COX enzymes [179]. R-ketorolac inhibitor was identified in a study on immortalized human ovarian adenocarcinoma cells (SKOV-3ip) and primary patient-derived ovarian cancer cells with similar effects to those of small molecule inhibitors of Rac1 (NSC23766) and Cdc42 (CID2950007/ML141). Eventually, selective inhibition of Cdc42 and Rac1 GTPases by R-ketorolac, an FDA-approved drug in racemic form was suggested to also be applied in humans [71]. Additionally, elevated expression and activity of Rac1 and Cdc42 was detected in tissues of ovarian cancer patients. Inhibition of Rac1 and Cdc42 in ovarian cancer patients can be achieved by the administration of racemic ketorolac, a mix of the R- and S-enantiomers. Contextually, it has been shown that perioperative use of ketorolac reduced ovarian cancer specific mortality [70]. Another study evaluated the effect of R-enantiomers on inhibition Rac1 and Cdc42. R-naproxen demonstrated the same activities against Rho-family GTPases Rac1 and Cdc42 as R-ketorolac in immortalized ovarian cancer (OvCa429, OvCa433) and cervical cancer (HeLa T4+) cells. Destabilization of nucleotide binding to Rac1 and Cdc42 is predicted to be due to rotational constraints and the position of carboxylate moieties of the R-enantiomers, which preferentially coordinate the magnesium ion [180]. Additionally, Cdc42 inhibition could represent a potent therapeutic target to various diseases [181]. The molecular probe ML 141, a selective inhibitor of Cdc42 GTPase, binds the guanine nucleotide-associated Cdc42 and induces ligand dissociation. Treatment with ML 141 effectively inhibited cell migration of ovarian carcinoma (A2780) cells even when using the smallest amount of the agent [182].

#### 3.2.2. NSC23766

Another therapeutic possibility is to block the Rho GTP formation by blocking the Rho GEFs and Rho interaction. The specific and dose-dependent inhibition of Rac1 activation by Rac-specific GEFs Trio or T-lymphoma invasion and metastasis 1 (Tiam1) is attributed to the Rac1 inhibitor NSC23766 [165]. Rac1 is considered as a potential target for anticancer therapy in ovarian cancer [183]. Chemical inhibition of Rac1 with NSC23766 reduced cellular proliferation of cervical cancer derived (C33A, SiHa) cell lines [133]. Moreover, rhein extracted from *Rheum palmatum* L. attenuated Rac1 and therefore inhibited migration and invasiveness of ovarian cancer (SKOV-3-PM4) cells through matrix metalloproteinases (MMPs) and Rac1/ROS/MAPK/AP-1 (Rac1/reactive oxygen species/mitogen-activated protein kinase/activating protein-1) pathway. The results of combinatory treatment with NSC23766 and rhein were consistent with NSC23766 alone [183].

#### 3.2.3. Berberine

Low doses of berberine derived from Coptidis rhizome inhibited Rho GTPase including RhoA, Cdc42, and Rac1 and cell migration in different human cancer cells. In addition, higher doses of berberine induced G2 arrest and apoptosis [184]. The anti-tumor effects of berberine were also observed in ovarian, cervical, and endometrial cancer model; however, the association with Rho GTPases or their components was not mentioned [185,186].

### 3.3. Farnesyltransferase Inhibitors and Geranylgeranyltransferase Inhibitor

The Ras pathway is essential for cell growth and proliferation. Farnesyltransferase inhibitors (FTIs) and geranylgeranyltransferase inhibitors (GGTIs) can disrupt the prenylation of oncogenic Ras. FTIs and GGTIs prevent Ras from binding to the membrane leading to Ras inactivation [187]. Lonafarnib, an FTI, blocked farnesylation with farnesyl pyrophosphate in SKOV3 and OVCAR5 cells leading to inhibition of cancer cell growth. A similar effect was observed after GGTI-298 treatment, which inhibited the geranylgeranylation with geranylgeranyl pyrophosphate in SKOV3 and OVCAR5 cells [188]. Moreover, R115777 (tipifarnib), an FTI, decreased tumor growth and cell turnover index (proliferation/apoptosis) in SKOV3 ovarian cancer cells [189].

### 3.4. Rho Regulators Inhibitors

Rho GTPase inhibitors revealed antitumor potential in different gynecological malignancies; however, their effects may have various side effects. After inhibition of Rho signaling pathways, especially in RhoA–C, Rac1, and Cdc42, it is very important to evaluate their effect on cellular processes, which can be associated with modulation in actin cytoskeleton, cell metabolism, or homeostasis in individuals [190]. Despite the mentioned side effects, Rho regulators (GEFs, GAPs, and GDIs) can attract more attention in cancer therapy, mainly due to higher specificity in regulation and signaling, which are necessary for proper Rho GTPase function [191,192,193].

Inhibition of GTPases can be achieved by Rho regulator inhibition. As mentioned above, Rac1 inhibitor NSC23766 inhibited Rac1 activation by Rac-specific GEFs Trio or Tiam1 [165] in ovarian [183] and cervical cancer [133]. Moreover, various Rho regulator inhibitors including high selective Cdc42 inhibitor ML141 [194], Scaff10-8 inhibitor of A-kinase anchoring protein (AKAP-Lbc)-mediated RhoA activation [195], inhibitor of Lbc-RhoA interaction [196], C21 chemical inhibitor of DOCK5 [197], and ITX3 inhibitor of the Trio/RhoG/Rac1 pathway [198], revealed their potential in cancer therapy; however, there is no their evidence regarding ovarian, cervical, and endometrial cancer.

**Figure 6:**
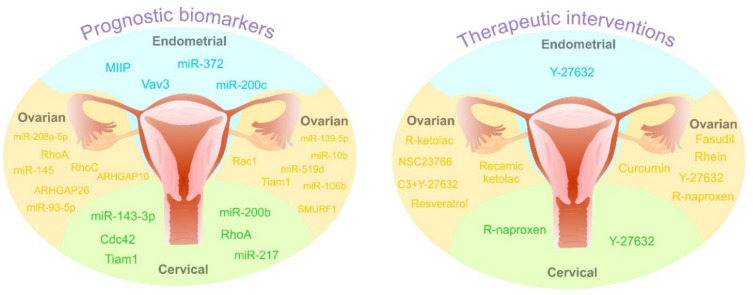
Prognostic value of Rho GTPases and their associated proteins or miRNAs in clinical and preclinical studies are shown on the left. Therapeutic interventions focused on Rho GTPases in different gynecological cancers are illustrated on the right. Abbreviations: RacGAP1, Rac GTPase activating protein; ARHGAP26, Rho GTPase-activating protein 26; ARHGAP10, Rho GTPase-activating protein 10; Tiam1, T-lymphoma invasion and metastasis 1; SMURF1, SMAD specific E3 ubiquitin-protein ligase 1; MIIP, migration and invasion inhibitory protein.

Targeting Rho GTPase components represents potential significance in ovarian, cervical, and endometrial cancer therapy, which could be further enhanced by identification of predictive tumor markers (Table 1). The graphical scheme of therapeutic interventions targeting Rho GTPases in gynecological cancers is illustrated in Figure 6.

## 4. Prognostic Value of Rho GTPases: Spectacular Biomarkers or “Blind Alley” of Personalized Medicine

The identification and validation of new markers in gynecological cancers based on molecular alteration of RNA, DNA, miRNA, and protein levels, in combination with clinical-pathological data, may improve early detection and contribute to personalized medicine as a treatment strategy of the 21st century [199,200]. Prognostic biomarkers predicting clinical outcomes, including the risk of recurrence or metastasis could help to determine an optimal therapy of malignancy after medications or surgical intervention [199]. The development of the “-omics” strategy has led to the characterization of novel markers of Rho GTPases, with differences in various stages of cervical, endometrial, and ovarian cancer, that are unique proteomic parameters that alone or in combination with other biomarkers could predict prognoses of these gynecological neoplastic transformations [201]. As described above, Rho GTPases are indispensable mediators of signal transduction regulating numerous cellular events including migration, survival, or proliferation, crucial for the maintenance of normal tissue. On the other hand, disequilibrium of these signaling pathways leads to cancer progression [2]. 

Horiuchi and colleagues analyzed the role of Rho GTPases (RhoA, RhoB, and RhoC) in various types of ovarian tumors including benign, malignant, and borderline tumors in fresh surgical specimens gathered from 42 women. The results proved an increase of RhoA and RhoC levels in malignant ovarian samples compared to benign tissues. Interestingly, a significant correlation was observed between higher levels of both mentioned Rho GTPases and serous carcinomas than those in other histological subtypes. Furthermore, the elevated mRNA expression of RhoA and RhoC was accompanied by higher tumor stages and metastatic spread [202]. Furthermore, in a retrospective study on 117 patients, Wang et al. focused on Rac GTPase activating protein (RacGAP1) and its expression level and activity in epithelial ovarian cancer. Acquired data confirmed an association of higher RacGAP1 expression with tumor grade, stage, and metastases. Patients with lower expression of the investigated protein had lower recurrence risk of disease and better overall survival rates compared to women with higher expression of RacGAP1 [203]. Additionally, Chen at al. studied Rho GTPase-activating protein 26 (ARHGAP26), a negative regulator of the Rho family, in a cohort of 85 ovarian cancer tissues and corresponding non-malignant regions. Data demonstrated a linkage between low expression rate of ARHGAP26 and poor prognosis with worse overall survival of patients [204]. Similarly, differences in the level of ARHGAP10 that inactivates Cdc42 were evaluated in patients (*n* = 75) with ovarian tumors. The results showed a decreased level of ARHGAP10 in 77.3% of analyzed malignant samples compared to non-malignant tissue regions. Moreover, a parallelism between the lower expression profile of ARHGAP10 inactivating of GTPase Cdc42 and lower overall survival compared to patients with higher expression of the activating protein was detected [205]. Furthermore, alterations in the expression level of Rac1 were found in a cohort of 150 specimens obtained from women with epithelial ovarian cancer. Survival analyses demonstrated differences in the expression profile of investigated Rac1 while a higher level of studied GTPase was associated with poor prognosis linked with early tumor recurrence in patients [206]. T-lymphoma invasion and metastasis 1 (Tiam1) acts as guanine nucleotide exchange factor (GEF) for proteins of the Rho family and its higher expression level was documented in various cancer types. 

Recently, Li and colleagues investigated cohorts of women with serous ovarian carcinoma (*n* = 182), borderline tumors (*n* = 76), and benign tumors (*n* = 72) focusing on Tiam1 expression and its possible correlation with patients’ prognosis. An abundance of studied Tiam1 was documented in a cohort of ovarian carcinoma (59.3%), while in groups of benign and borderline tumors, higher expression was only identified in 12.5% and 31.6%, respectively. An increased Tiam1 expression was closely related to the clinical stage, histological grade, and lower overall survival rates. The results suggest that Tiam1 can serve as a molecular prognostic marker for patients with ovarian cancer [207]. Interestingly, Wang et al. analyzed SMAD specific E3 ubiquitin-protein ligase 1 (SMURF1) regulating various cellular processes. The correlation of SMURF1 overexpression and RhoA/ROCK signaling pathways promoting metastasis was observed in ovarian cancer cell lines (OVCAR3). Moreover, an increased level of SMURF1, which was detected in 80 ovarian serous cystadenocarcinoma samples, was accompanied by a shorter overall survival of patients with ovarian cancer [208].

Alterations in individual Rho GTPases and associated proteins are potential prognostic biomarkers also in other gynecological malignancies. A study analyzing 162 cervical squamous cell carcinoma tissues and 33 normal tissue specimens demonstrated a strong correlation between carcinoma tissue and a high level of Cdc42 associated with the clinical stage of patients compared to controls [134]. Moreover, the higher expression of RhoA could play an important role in the prediction of distant metastasis after concurrent chemoradiotherapy in bioptic samples obtained from 49 cervical cancer patients suggested the key role of RhoA as a prognostic marker [209]. In addition, experimental data evaluating 46 samples of cervical squamous cell carcinoma and 34 cervicitis tissues (control samples) exhibited an association between raised RhoA level and vascular invasion and metastasis [132]. Yang et al. examined Tiam1 protein expression in 174 cervical cancer samples, 92 cervical intraepithelial neoplasia, and 32 normal cervical tissues. The study demonstrated high expression of Tiam1 in cervical cancer specimens compared to cervical intraepithelial neoplasia and normal cervical tissues. Furthermore, overexpression of Tiam1 was associated with advanced clinical stages, metastasis, HPV infection, and overall survival of patients [210].

Rho GTPase signaling pathways are also obviously connected with the progression of endometrial cancer (EC), and their fluctuations on molecular and proteomic levels may be used in the investigation and routine application of these signatures as prognostic biomarkers [211]. However, evidence concerning Rho GTPases’ prognostic abilities in the clinical sphere is insufficient. An association between the upregulation of migration and invasion inhibitory protein (MIIP), activation of Rac1, and cell migration of EC cell lines was determined. An evaluation of the MIIP levels in 205 EC patient samples, 63 with atypical hyperplasia and 116 with normal tissue, revealed that EC patients had elevated expression of MIIP associated with an advanced stage of the disease and lymph node metastasis [94]. Furthermore, another study analyzed the expression of Vav3 acting as a guanine nucleotide exchange factor regulating Rho/Rac signaling pathways in tissue collected from women with diagnosed EC and healthy controls. Here, overexpression of Vav3 was seen in cancer samples but a correlation with clinical-pathological features and prognostic outcomes of patients was not detected [212].

Several miRNAs, small non-coding RNAs, have the ability to alter cell proliferation, migration, and invasion via affecting ROCK signaling, suggesting their significant prognostic potential in gynecological cancers. RhoC was observed as the target by miR-106b transfection, which suppressed tumor development and progression of epithelial ovarian cancer xenograft mouse models [213]. Furthermore, overexpression of miR-93-5P inhibited tumorigenesis and progression via targeting RhoC in epithelial ovarian cancer (OVCAR3, SKOV3/DDP, HO8910-PM) cells and OVCAR3 xenografts [214]. Another study showed that decreased HOXD10 expression caused by miR-10b overexpression might trigger synthesis of pro-metastatic molecules, such as RhoC and MMP14 and thus contribute to the acquisition of metastatic phenotypes in different epithelial ovarian cancer cell lines [215]. Kim et al. described that miR-145 represents tumor suppressor in ovarian cancer: it downregulates ROCK-1 through the ROCK-1/NF-κB signaling pathway and directly targets HMGA2 oncoprotein translocated then to the nucleus in a ROCK-dependent manner. It seems that low miR-145 expression represents a good biomarker of poor prognosis of ovarian carcinoma and miR-145 may serve as a predictor of patient outcomes [216]. Sang and colleagues found that tumor suppressive miR-519d or siRhoC co-transfection reversed E2F1 oncogenic effects in ovarian cancer cells (increased RhoC, Bcl-2, cyclin D1, survivin, MMP2, MMP9, STAT3, and HuR) [217]. Downregulation of miR-139-5p significantly correlates with stage, lymph mode metastasis, and poor overall survival of patients with ovarian cancer. It was documented that miR-139-5p plays an important role as a tumor suppressor in ovarian cancer through direct binding to ROCK-2, thus providing a novel molecular prognostic marker [218]. Increased expression of miR-208a-5p suppressed the migration and invasion of OVCAR-3 cells, however, this effect can be reversed by DAAM1 overexpression. Authors documented that inhibition of DAAM1 prevents Wnt/Fz activation of Rho signaling and summarized that miR-208-5p/DAAM1 axis may be a novel prognostic marker/clinical target in metastatic phenotype of ovarian cancer [219].

MiRNAs have also been described as potential prognostic markers in endometrial and cervical cancers. Anti-oncogenic miR-372 suppressed tumorigenesis and the development of endometrial cancer xenografts and HEC-1B cells via targeting the RhoC expression [220]. MiRNA200c functions as a tumor-suppressor, which reduces stem cell-like characteristics, EMT phenotype and aggressive behavior, and increases sensitivity to taxanes [221]. Mechanistic analyses using endometrial cancer cells (HEC50, AN3CA) showed that tumor-suppressive activities of miRNA200c are executed by suppression of mesenchymal and neuronal genes expression (Rho GTPase activating protein 19 (ARHGAP19), fibronectin 1, moesin, neurotrophic tyrosine receptor kinase type 2, and leptin receptor) that are included in cell motility and anoikis resistance and by targeting ZEB1/2 (facilitates restoration of E-cadherin expression). In addition, miR-200b inhibited the RhoE function and the epithelial–mesenchymal transition in cervical cancer (HeLa) cells [222]. Overexpression of tumor suppressor miR-217 reduced colony formation and cell invasion and significantly increased apoptosis in cervical cancer (SiHa, HeLa) cells through ROCK-1 targeting [223]. Recently, Song et al. described that miR-143-3p, by interaction with OIP5-AS1 (long non-coding RNA), positively regulated cervical cancer progression through an increase in ROCK-1 expression [224]. Contextually, the identification of specific molecules of Rho GTPase signaling pathways and associated interactions may have potential in the determination of predictive or prognostic markers. On the other hand, therapeutic biomarkers are undoubtedly needed in gynecological cancer management as crucial parts of current and future personalized medicine [225]. Despite limited evidence, RhoB [226] and RhoC [227] as well as other Rho GTPase associated interactions, including melanoma cell adhesion molecule (MCAM) [228] or regulator of chromosome condensation 2 (RCC2) [229], represent biomarkers with potential in gynecologic cancer therapy. Table 2, as well as Figure 6, summarize the prognostic role of Rho GTPases, associated proteins, and miRNAs in ovarian, cervical, and endometrial malignancies.

## 5. Towards Predictive, Preventive, and Personalized Medical (PPPM/3PM) Approaches in Gynecological Oncology: Prominent Examples Involving Rho GTPases as a Target 

The steadily increasing number of chronically diseased individuals altogether burdens population health and the health economy worldwide. The “3PM” approach has been proposed to be an optimal and the most cost-effective option for healthcare systems [230,231]. However, the paradigm change from reactive to predictive, preventive, and personalized medicine demands clear implementation strategies and powerful tools discussed below and exemplified by gynecological oncology.

### 5.1. Rho GTPases in Prediction of Aggressive Gynecologic Cancers and Metastatic Disease: Prominent Examples

As demonstrated above, Rho GTPases are highly specific for tumor stem cells and tumor stroma. They are crucial for growth and survival of tumor cells, cell migration, cancer cell invasion, and formation of metastases [232]. For example, DAAM1-small G protein RhoA axis plays a vital role in aggressive metastatic ovarian and breast cancers, presenting an excellent diagnostic and therapeutic target [219].

Further, early and predictive diagnostics of breast cancer in young populations are particularly challenging [199]. Further, the disease is frequently more aggressive for young women. However, currently applied mammography-based screening programs are focused on 40+ years-old population, whereas tools essentially needed for screening in young (18+ years of age) female populations are missing that is particularly problematic for the pregnancy-associated breast cancer and other aggressive disease sub-types [233].

#### 5.1.1. Rho GTPases as a Target for Early Detection of Pre/Cancerous Lesions in High Mammographic Density Breast

To cover these evident deficits, the protein cluster of Rho GTPases might be an excellent target for early detection of pre/cancerous lesions in high mammographic density breasts associated with an increased risk of breast cancer. In a study by Lisanti et al. “breast density” gene transcripts’ signature was generated. The results indicate hyper-activation of several key signaling pathways; Rho GTPases JNK1-related stress signaling is hyper-activated in the high density breast and the breast tumor stroma connecting fibrosis, inflammation, and stemness for cancer prevention [234].

#### 5.1.2. Predictive Diagnosis of Breast Cancer Based on RhoA Patterns: Multiomics Approach

**Figure 7:**
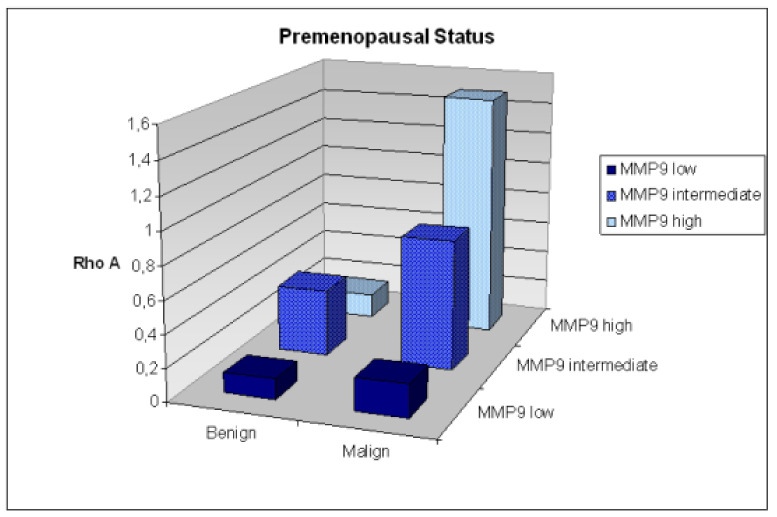
Precise patient stratification for breast cancer prediction based on RhoA expression patterns in circulating leucocytes; example is provided on premenopausal patients [235].

Predictive diagnosis of breast cancer in predisposed individuals—both premenopausal and postmenopausal—is possible based on multiomic approach involving RhoA. To this end, expression patterns of RhoA in circulating leucocytes have been demonstrated as being shifted in breast cancer patients versus healthy controls and, therefore, being reflective for a disease development. However, predictive diagnosis-relevant differences in corresponding patterns have only been demonstrated in multiomic approach application. Indeed, patients stratified by both – menopausal status and high activity level of metalloproteinase 9 in blood serum were simply identifiable against controls (see Figure 7) [235]. Contextually, multi-level diagnostics including multiomic approach are decisive for clinically relevant risk assessment in breast cancer [236] and in cancer prediction generally [237]. It is well justified that DNA-based analysis alone does not provide sufficient information for accurate diagnosis and treatment of cancer. Therefore, a gradual shift from a “genetic disease” to a “metabolomic disorder” is concluded for an advanced understanding and management of cancer. Consequently, hybrid technologies such as metabolomic tools and a combination of several omics are considered to advance early and predictive diagnostics [238].

Considering the etiology of multi-factorial oncologic diseases, phenotyping plays a key role in multi-level diagnostics and patient stratification as recently proposed for particularly aggressive cancer subtypes such as triple-negative premenopausal breast cancer [239,240].

### 5.2. Targeted Prevention

Based on the precise prediction of the disease, cost-effective targeted prevention is implementable. Contextually, for oncologic diseases Rho GTPases is an excellent target. The arguments are summarized below.

#### 5.2.1. Inflammation

On one hand, chronic inflammation is a well-acknowledged attribute of pre/cancerous lesions [241]. On the other hand, upregulation of the RhoA/ROCK signaling pathway is known to cause increased inflammation, immune cell migration, and cell adhesion [242]. Consequently, Rho kinase inhibitors have been proposed as highly potent targets for cancer prevention and treatment [243].

#### 5.2.2. Chronic Wounds and Impaired Healing

On one hand, chronic inflammation and impaired healing are clear indicators for a predisposition to cancer development [241,244]. On the other hand, deregulation of Rho-ROCK signaling network involved in wound healing contributes to associated chronic inflammation and impaired healing resulting in the formation of a microenvironment for aggressive tumor progression. Consequently, technological approaches have been suggested to target the Rho-ROCK pathway for cancer prevention and therapeutic benefits regulating tumor microenvironment [245].

#### 5.2.3. Rho GTPases-Based Prevention of Endometrial Carcinoma in Obese Phenotype

Obesity and diabetes mellitus type 2 are both highly relevant for developing several gynecological cancers such as endometrial carcinomas, among others. To this end, activation of NMU pathway in endometrial cancer cells linked to various adhesion molecules, (CD44, integrin alpha1, etc.), production of extracellular matrix ligands (hyaluronan and collagen IV) as well as upregulation of SRC, Rho A, and Rac1 have been demonstrated [111]. In this way, the signaling-SRC-Rho GTPase axis is activated in endometrial cancer cells, changing cell motility and proliferation. Consequently, the NMU signaling SRC-Rho GTPase cascade is an excellent target for preventing endometrial cancer development in obese phenotype.

#### 5.2.4. Small GTPases are Regulated by Nutrients—An Approach for Dietary Cancer Prevention

Adipose tissue plays a crucial role in obesity-related pathologies such as accelerated aging, chronic inflammation, impaired healing, diabetes type 2, and several cancer types. Rho GTPases are directly involved in obesity-related pathologic cascades [246]. On the other hand, small GTPases are regulated in response to nutrients that open the door for targeted dietary measures to prevent secondary complications in overweight and obese patients [247].

#### 5.2.5. Targeted Chemoprevention

Application of dietary phytochemicals is a promising approach for targeted chemoprevention of cancer and metastatic disease [248,249]. Phenolics, flavonoids, and anthocyanins are phytochemicals abundant in many natural products such as berries and their extracts. Their antioxidant, antimicrobial, anti-inflammatory, anti-diabetic, neuroprotective, hepatoprotective, and cardioprotective effects have been demonstrated to target directly the small GTPases and the PI3K/AKT pathway [250]. Further, saponins extracted from the byproduct of *Asparagus officinalis* L. have been demonstrated to target Rho GTPase signaling pathway suppressing tumor cell migration and invasion [251]. Similar effects have been demonstrated for paeoniflorin which inhibits hepatocyte growth factor-induced migration, invasion, and actin rearrangement via suppression of c-Met-mediated RhoA/ROCK signaling [252].

Targeted chemoprevention is applicable, for example, to individuals in suboptimal health conditions who are strongly predisposed to cancer development as stated in recently published studies [253,254].

### 5.3. Personalized Treatment Algorithms

Personalized treatment algorithms are usually based on individualized patient profiling which includes phenotyping and multiomics molecular signature characteristic for pathology or predisposition to it. Contextually, optimal disease modeling plays a crucial role in creating targeted treatment algorithms [255]. As detailed above, Rho GTPases are one of the best targets for personalization of treatments as the key protein cluster involved in a cancer development and progression. Contextually, based on the function of Rho GTPases in a variety of both physiologic and patho-physiologic processes, the treatment opportunities include

- Personalized preventive measures applied to suboptimal health;
- Personalized primary cancer prevention and treatments at the level of pre-cancerous lesions;
- Personalized treatment of cancer;
- Personalized secondary prevention (e.g., prevention of cancer in obese and diabetic patients);
- Personalized prevention of metastatic disease [256,257].

## 6. Conclusions

Deregulation of the Rho GTPase function is involved in various pathological processes including tumorigenesis, cancer progression, metastatic disease, and therapy resistance.

This review provides the link between Rho GTPases signaling pathways that collectively contribute to biologic and pathologic mechanisms involved in gynecologic cancers at the level of cancer cell growth, invasiveness, metastatic spreading, and therapeutic response. Numerous different genetic and epigenetic mechanisms can deregulate the Rho GTPase signaling networks in gynecologic tumors, which include in/activating intact or mutated Rho GTPases within regulation cascades as well as changes in their expression patterns that altogether modulates signaling with pro- or anti-tumorigenic effects depending on the cell context and tumor type. The fact that cancer cell proliferation and invasion can be affected by disrupting the interaction between participating factors in Rho signaling pathways provides a rationale for developing pharmacological compounds to prevent malignancies and to establish advanced personalized treatment algorithms with effective therapeutic agents/targets. This approach is based on individualized patient profiling which includes phenotyping and multiomics molecular signature characteristic for pathology or predisposition to it, thus improving individual outcomes. Unraveling these networks will enable the development of compounds that could be therapeutically or screening effective.

## Figures and Tables

**Table 1:** Targeting of Rho GTPase components in gynecologic cancer treatment.

Therapeutics	Cancer Types	Target	Reference
Y-27632	Cervical CaSki cells	↓ ROCK-1, ↑ RhoC	[170]
	Cervical SiHa cells	↓ ROCK-2	[171]
	Ovarian CAOV-3, PA-1 cells	↓ LPA	[173]
	Endometrial HEC-1A cells	Reversed effect on LPA and calpeptin-induced cell proliferation	[174]
C3 + Y-27632	Ovarian OVCAR3, SKOV-3, CAOV-3 cells	↓ leptin-induced uPA	[172]
Fasudil	Ovarian SKOV-3ip1 cells and xenografts	↓ intracellular cytoskeletal rearrangement, ↓ tyrosine phosphorylation of paxillin, ↓ serine phosphorylation of myosin light chain	[176]
	Ovarian A2780 cells	Modulation of HIF-1α	[166]
NSC23766	Cervical C33A, SiHa cells	↓ Rac1	[133]
Rhein	Ovarian SKOV-3-PM4	Modulation of MMPs and Rac1/ROS/MAPK/AP-1	[183]
R-ketorolac	Ovarian SKOV-3ip and primary patients derived ovarian cancer cells	Similar effect as NSC23766 and CID2950007/ML141	[71]
Racemic ketorolac	Ovarian cancer patients	↓ Rac1, ↓ Cdc42	[70]
R-naproxen	Ovarian OvCa429, OvCa433, cervical HeLa T4 + cells	↓ Rac1, ↓ Cdc42	[180]
ML-141	Ovarian A2780 cells	↓ Cdc42	[182]
Curcumin	Ovarian SKOV-3 cells	Activation of RhoA/Rho-kinase signaling	[177]
Resveratrol	Ovarian OVCAR-3 cells	↓ *ARH-I*, ↓ LC3-positive autophagic vacuoles formation	[178]

Explanatory notes: ↑, induction/increase; ↓, inhibition/decrease. Abbreviations: LPA, lysophosphatidic acid; uPA, urokinase plasminogen activator; HIF-1α, hypoxia-inducible factor-1α; MMP, matrix metalloproteinase; Cdc42, cell division cycle protein 42; ROCK, Rho-kinase; Rho, member of GTPases family; Rac, member of Rho family of GTPases; ROS, reactive oxygen species; MAPK, mitogen-activated protein kinase; AP-1, activating protein-1.

**Table 2:** Prognostic value of Rho GTPases in gynecological cancers.

Type of Cancer	Type of Study	Prognostic Markers	Effect	References
	clinical trial (*n* = 42)	↑ RhoA, RhoC	higher tumor stages, metastasis spreading	[201]
	clinical trial (*n* = 117)	↓ RacGAP1	lower risk of recurrence, better overall survival	[203]
	clinical trial (*n* = 85)	↓ ARHGAP26	lower overall survival, poor prognosis	[204]
	clinical trial (*n* = 75)	↓ ARHGAP10	lower overall survival	[205]
	clinical trial (*n* = 150)	↑ Rac1	poor prognosis, risk of recurrence	[206]
	clinical trial (*n* = 330)	↑ Tiam1	association with clinical stage, histological grade, lower overall survival	[207]
	in vitro (OVCAR3) clinical study (*n* = 80)	↑ SMURF1	lower overall survival	[208]
	in vivo (BALB/C nude mice)	↑ miR-106b	suppression of tumor development and progression	[213]
OC	in vitro (OVCAR3, SKOV3/DDP, HO8910-PM) in vivo (BALB/C nude mice)	↑ miR-93-5p	suppression of tumorigenesis and cancer progression	[214]
	in vitro (JHOC-5, JHOC-7, JHOC8, JHOS-2, JHOS-3, JHOS-4, JHOM-1, OVCAR3)	↑ miR-10b	acquisition of metastatic phenotypes	[215]
	clinical trial (*n* = 74)	↓ miR-145	poor prognosis	[216]
	in vitro (SKOV3, HO8910, ES-2, CAOVR3, OVCAR3, A2780 and A2780PTX)	↑ miR-519d	reversion of oncogenic effect of E2F1	[217]
	in vitro (OVCAR3)	↑ miR-208a-5p	suppression of cell migration and invasion	[219]
	clinical trial (*n* = 46)	↓ miR-139-5p	correlation with FIGO stage, lymph node metastasis, poor overall survival	[218]
CC	clinical trial (*n* = 195)	↑ Cdc42	association with clinical stage of tumors	[134]
	clinical trial (*n* = 49)	↑ RhoA	prediction of distant metastasis after chemotherapy	[209]
	clinical trial (*n* = 80)	↑ RhoA	vascular invasion and metastasis	[132]
	clinical trial (*n* = 298)	↑ Tiam1	advanced clinical stage, metastasis spreading, HPV infection, poor overall survival	[210]
	in vitro (SiHa, HeLa)	↑ miR-217	reduction colony formation, invasion, increased apoptosis	[223]
	in vitro (HeLa)	↑ miR-200b	inhibition of EMT	[222]
	in vitro (C33)	↑ miR-143-3p	positive regulation of cancer progression	
EC	clinical trial (*n* = 364)	↑ MIIP	advanced clinical stage, lymph node metastasis	[94]
	clinical trial (*n* = 265)	↑ Vav3	no significant with clinic pathological features and prognostic outcomes for patients	[212]
	in vitro (HEC-1B) in vivo (BALB/C nude mice)	↑ miR-372	suppression of tumorigenesis	[220]
	in vitro (HEC50, AN3CA)	↑ miR-200c	inhibition of cell motility and anoikis resistance	[221]

Explanatory notes: ↑ increase; ↓ decrease Abbreviations: CC, cervical cancer; EC, endometrial cancer; OC, ovarian cancer; Cdc42, cell division cycle protein 42; RacGAP1, Rac GTPase activating protein; ARHGAP26, Rho GTPase-activating protein 26; ARHGAP10, Rho GTPase-activating protein 10; Tiam1, T-lymphoma invasion and metastasis 1; SMURF1, SMAD specific E3 ubiquitin-protein ligase 1; MIIP, migration and invasion inhibitory protein; EMT, epithelial–mesenchymal transition.
